# Degradation of oxytetracycline in wastewater by catalytic ozonation with eggshell-derived calcium peroxide

**DOI:** 10.1039/d5ra06601h

**Published:** 2025-10-28

**Authors:** Apiradee Sukmilin, Piyapong Pankaew, Jaroenporn Chokboribal, Chalor Jarusutthirak

**Affiliations:** a Faculty of Science and Technology, Phranakhon Rajabhat University Bangkok 10220 Thailand Apiradee@pnru.ac.th; b Division of Industrial Materials Science, Faculty of Science and Technology, Rajamangala University of Technology Phra Nakhon Bangkok 10800 Thailand; c Materials Science Program, Faculty of Science and Technology, Phranakhon Rajabhat University Bangkok 10220 Thailand; d Department of Environmental Technology and Management, Faculty of Environment, Kasetsart University Bangkok 10900 Thailand

## Abstract

Calcium peroxide (CaO_2_) was successfully synthesized from calcium-rich eggshell waste through a multi-step process. First, the eggshell waste was naturally dried, ground, and calcined at varying temperatures (700, 800, or 900 °C) for 2 hours, resulting in the formation of calcium oxide (CaO). Subsequently, CaO_2_ was synthesized *via* a precipitation method, in which CaO was mixed with hydrogen peroxide (H_2_O_2_) at varying concentrations (25%, 30%, or 35%). The formation of CaO_2_ was initially confirmed by its characteristic yellowish appearance. The properties of the eggshell waste, CaO, and CaO_2_ were characterized using X-ray diffractometry (XRD), Fourier-transform infrared spectroscopy (FTIR), and scanning electron microscopy (SEM). The XRD results indicated that higher calcination temperatures enhanced the crystallinity of CaO, while increasing the H_2_O_2_ concentration led to a reduction in the crystalline structure of CaO_2_. The performance of CaO_2_ as a catalyst in catalytic ozonation was evaluated for the degradation of oxytetracycline (OTC) in synthetic wastewater. Under the designated conditions (pH 7, 3 g per L CaO_2_, and 60 min reaction time), OTC removal efficiency reached 100%, whereas sole ozonation achieved only 85.7%. The pseudo-first-order reaction rate constant (*k*_obs_) for composite B (30% H_2_O_2_ with 1 : 1 mole ratio between CaO and H_2_O_2_) was 0.1152 min^−1^, which was significantly higher than that of sole ozonation (*k*_obs_ = 0.0365 min^−1^), demonstrating the catalytic efficiency of CaO_2_.

## Abstract 2

Calcium peroxide synthesized from eggshell waste effectively acted as a catalyst with ozone. Oxytetracycline degradation reached ∼100% with CaO_2_, compared to 85.72% with ozone. The novelty lies in explaining the mechanism *via* Lewis acid–base theory.

## Graphical abstract

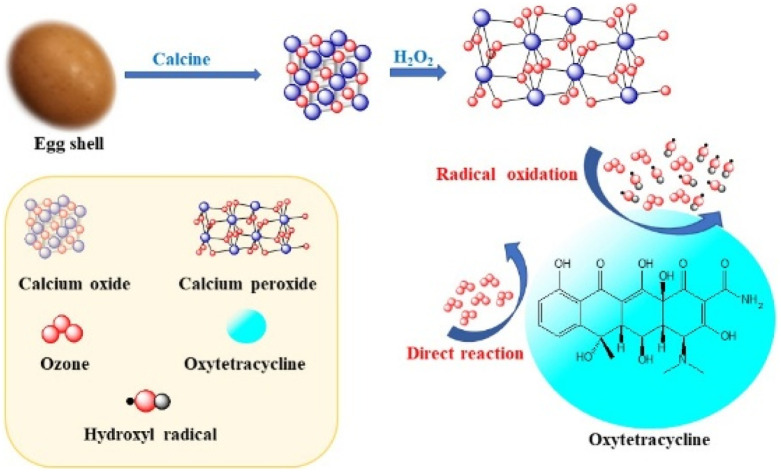

## 1. Introduction

Calcium peroxide (CaO_2_) has garnered increasing attention in wastewater treatment due to its versatility as an oxidant and controlled-release source of hydrogen peroxide (H_2_O_2_).^1^ As a thermally stable inorganic peroxide, the hydrolysis of CaO_2_ generates both H_2_O_2_ and calcium hydroxide (Ca(OH)_2_), facilitating its application in both biological and chemical treatment processes.^2^ In activated sludge processes, CaO_2_ has been used as an oxygen source, enhancing microbial degradation of organic pollutants while reducing energy-intensive aeration requirements.^3^ In addition, it has demonstrated high efficiency in heavy metal removal, where the combined effects of H_2_O_2_ oxidation and Ca(OH)_2_ precipitation enable the removal of heavy metals (Pb, Cu, Zn, Ni, Cd, As).^4,5^ Furthermore, CaO_2_ plays a crucial role in advanced oxidation processes (AOPs), particularly in the degradation of recalcitrant contaminants such as dyes,^6,7^ phenol,^8^ and emerging contaminants,^9–11^ through the generation of hydroxyl radicals (OH˙). Several studies have explored its application as a catalyst in the ozonation process, wherein its controlled dissolution facilitates the gradual release of H_2_O_2_.^7,8^ This synergistic effect has been reported to improve the degradation of pharmaceutical products, such as sulfonamide,^9^ metronidazole,^10^ sulfamethoxazole,^11^ oxytetracycline,^12^ sulfolane,^13^ and diclofenac,^14^ achieving removal efficiencies of up to 80%. Furthermore, the alkaline conditions induced by Ca(OH)_2_ accelerate ozone decomposition into reactive species, further enhancing pollutant degradation. Its environmentally friendly nature, cost-effectiveness, and ease of handling compared to other oxidants, make CaO_2_ a promising alternative for sustainable wastewater treatment.^7,10,11^

The synthesis of CaO_2_ in the presence of H_2_O_2_ has been studied extensively, with various calcium precursors, including calcium chloride (CaCl_2_), calcium hydroxide (Ca(OH)_2_), calcium nitrate (Ca(NO_3_)_2_), and calcium sulfate (CaSO_4_).^15–17^ However, the high cost and environmental concerns associated with conventional precursors have driven interest in sustainable alternatives. Eggshell waste, an abundant calcium-rich byproduct, has emerged as a promising raw material for CaO_2_ synthesis due to its high calcium carbonate (CaCO_3_) content.^18^ Eggshell waste was chosen because it is abundant, cost-effective, and environmentally friendly, providing a low-cost raw material while reducing waste and adding environmental and economic value. Utilizing eggshell waste as a precursor not only provides a cost-effective and environmentally friendly alternative but also aligns with circular economy principles by mitigating waste disposal issues associated with large-scale hatcheries.^18,19^ The synthesis of CaO_2_ from eggshell waste using precipitation typically involves a two-step process: thermal decomposition of CaCO_3_ to calcium oxide (CaO) at temperatures exceeding 700 °C, followed by the reaction of CaO with H_2_O_2_ to form CaO_2_.^20,21^ Studies have demonstrated that complete conversion of CaCO_3_ to CaO occurs at approximately 900 °C, underscoring the critical role of temperature in optimizing CaO formation.^20,21^ Despite the potential of CaO_2_ synthesis from eggshell waste, published research on the topic remains limited, particularly regarding optimizing key synthesis parameters such as calcination temperature, H_2_O_2_ concentration, and the Ca(OH)_2_-to-H_2_O_2_ molar ratio. These factors substantially influence the properties of the synthesized CaO_2_, which in turn determine its performance in environmental applications, particularly in catalytic ozonation.^22^

Among emerging contaminants in water systems, antibiotics such as oxytetracycline (OTC) pose major environmental risks due to their extensive use in aquaculture, animal husbandry, and agriculture.^23,24^ Often, conventional wastewater treatment processes are insufficient for complete OTC degradation, leading to its persistence in aquatic environments and contributing to the proliferation of antibiotic resistance.^25^ While ozonation is widely used for OTC removal, often its efficiency is hindered by the formation of toxic by-products.^25,26^ AOPs that combine ozone (O_3_) with H_2_O_2_ have demonstrated enhanced OTC degradation by generating OH˙ radicals.^12,27^ However, direct H_2_O_2_ addition presents challenges related to rapid decomposition and handling difficulties. As a controlled-release H_2_O_2_ source, CaO_2_ offers a more stable and efficient alternative in catalytic ozonation.^28^ Despite the promising potential of CaO_2_ synthesized from eggshell waste as a catalyst in ozonation, its application in OTC degradation remains unexplored. Furthermore, no published research has systematically investigated the effects of synthesis parameters, including calcination temperature, H_2_O_2_ concentration, and molar ratio, on the physicochemical characteristics and catalytic efficiency of eggshell-derived CaO_2_ in ozonation applications.

**Fig. 1:**
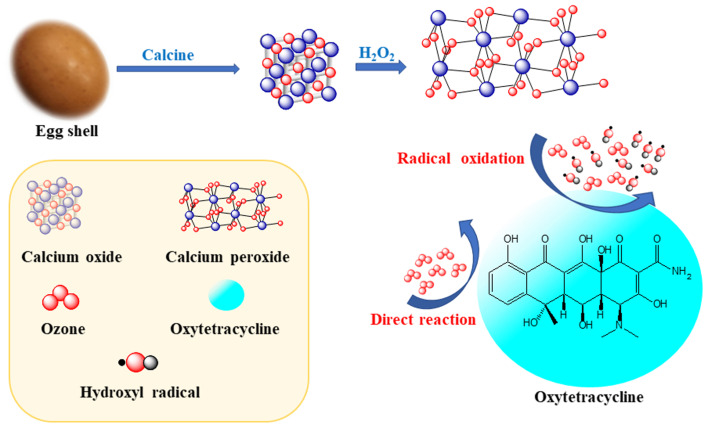
Schematic summary of the methodology and results of present study.

Thus, the current study aimed to synthesize CaO_2_ from eggshell waste using a precipitation method while systematically optimizing key synthesis parameters: calcination temperature, H_2_O_2_ concentration, and the Ca(OH)_2_-to-H_2_O_2_ molar ratio. The physicochemical properties of the synthesized CaO_2_ were characterized using X-ray diffractometry (XRD) and Fourier-transform infrared spectroscopy (FTIR). Then, the catalytic efficiency of CaO_2_ in ozonation was evaluated based on batch experiments using OTC as a model antibiotic. This research should contribute to the development of sustainable and efficient strategies for the treatment of OTC or other antibiotic contaminants, or both, in wastewater systems. The methodology and overview of the present study are summarized in Fig. 1.

## 2. Experimental

### 2.1. Materials

The collected eggshell waste was dried at 100 °C for 2 hours, then crushed into small pieces and stored in a desiccator before use. This experiment used analytical-grade reagents, including oxytetracycline (Thermo Fisher Scientific), hydrogen peroxide (35%, Chem-Supply), sodium hydroxide (Ajax), and sulfuric acid (RCI Labscan). Commercial calcium peroxide (30%, STP Chem Solution) was used as a reference for comparison with the synthesized calcium peroxide from the eggshell waste. Sodium thiosulfate (Kemaus) was used to quench residual ozone. All solutions were prepared using deionized water.

### 2.2. Preparation of CaO_2_ from eggshell waste

**Fig. 2:**
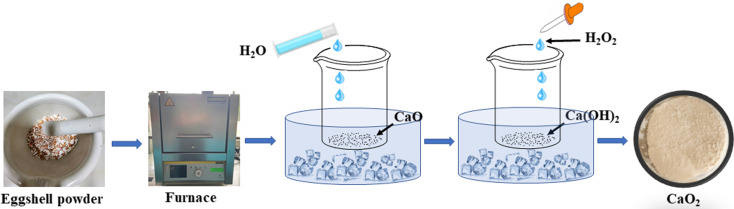
Schematic diagram of calcium peroxide (CaO_2_) synthesis from eggshell waste.

CaO_2_ was synthesized from eggshell waste using a calcination and precipitation method,^20^ as shown in Fig. 2. The eggshell waste was calcined at 700 °C, 800 °C, or 900 °C for 2 hours. After calcination, the resulting CaO was added to water under an ice bath to form Ca(OH)_2_. The concentration of H_2_O_2_ was varied (25%, 30%, or 35%), with different Ca(OH)_2_-to-H_2_O_2_ molar ratios (1 : 1, 7 : 1, 8.5 : 1, or 10 : 1), as shown in Table 1. The formation of CaO_2_ was indicated by the appearance of a yellowish slurry. The synthesized CaO_2_ was characterized using XRD, FTIR, and SEM. The final product was stored in a desiccator prior to use.

**Table 1:** H_2_O_2_ concentrations and Ca(OH)_2_-to-H_2_O_2_ molar ratios used in CaO_2_ synthesis

Composite	Concentration of H_2_O_2_ (%)	Molar ratio (Ca(OH)_2_ : H_2_O_2_)
A	25	1 : 1
B	30	1 : 1
C	35	1 : 1
D	25	7 : 1
E	30	8.5 : 1
F	35	10 : 1

### 2.3. Characterization of eggshell waste, CaO, and CaO_2_

The eggshell waste, calcined eggshell waste, and synthesized calcium peroxide were characterized to determine their structural, chemical, and morphological properties using XRD, FTIR, and SEM, as detailed below.

#### 2.3.1. X-ray diffraction

X-ray Diffraction (XRD) was performed to identify the crystalline phases of the samples. The XRD data were collected using a Philips X'Pert-MPD X-ray diffractometer (PW 3020 vertical goniometer and PW 3710 MPD control unit) with Bragg–Brentano para-focusing optics. The diffraction patterns were recorded in the 2*θ* range of 10–70° with a scanning rate of 2° min^−1^. The phase composition of the samples was determined using the direct peak intensity comparison method.^29^

#### 2.3.2. Fourier transformed infrared spectroscopy

Fourier-transform infrared spectroscopy (FTIR) analysis was conducted to identify the functional groups present in the samples. The spectra were recorded using a PerkinElmer FTIR spectrometer in attenuated total reflectance (ATR) mode with a resolution of 4 cm^−1^ over the wavenumber range 4000–500 cm^−1^. Prior to analysis, the samples were dried at 100 °C overnight to remove residual moisture.

#### 2.3.3. Scanning electron microscopy

Scanning electron microscopy (SEM) analysis was performed to examine the surface morphology and elemental composition of the samples. Images were captured using a JEOL-JSM 5600 LV microscope equipped with a 6587 energy-dispersive X-ray spectroscopy (EDS) detector at an accelerating voltage of 15 kV. The samples were mounted on a sample holder using adhesive carbon foil and sputter-coated with gold to enhance conductivity.

### 2.4. Investigation of catalytic ozonation efficiency

The catalytic ozonation experiments were conducted to evaluate the efficiency of synthesized calcium peroxide for the degradation of oxytetracycline (OTC). The ozonation system was operated at ambient temperature, with ozone generated from dry air using an ozone generator. In the catalytic ozonation process, synthesized calcium peroxide was introduced into the reactor. The initial OTC concentration was set at 5 mg L^−1^, with a solution pH of 7 and a calcium peroxide dosage of 3 g L^−1^. Unreacted ozone was trapped using a 2% potassium iodide solution. The residual OTC concentration was measured using a UV-Vis spectrophotometer at 272 nm. All experiments were conducted in triplicate, with the results presented as average values from three independent measurements, with results shown in figures and tables. Sole ozonation (without calcium peroxide) was performed as a control experiment under identical conditions to compare its efficiency with catalytic ozonation.

## 3. Results and discussion

### 3.1. Characterization of eggshell waste, CaO, and CaO_2_

#### 3.1.1. X-ray diffraction analysis

**Fig. 3:**
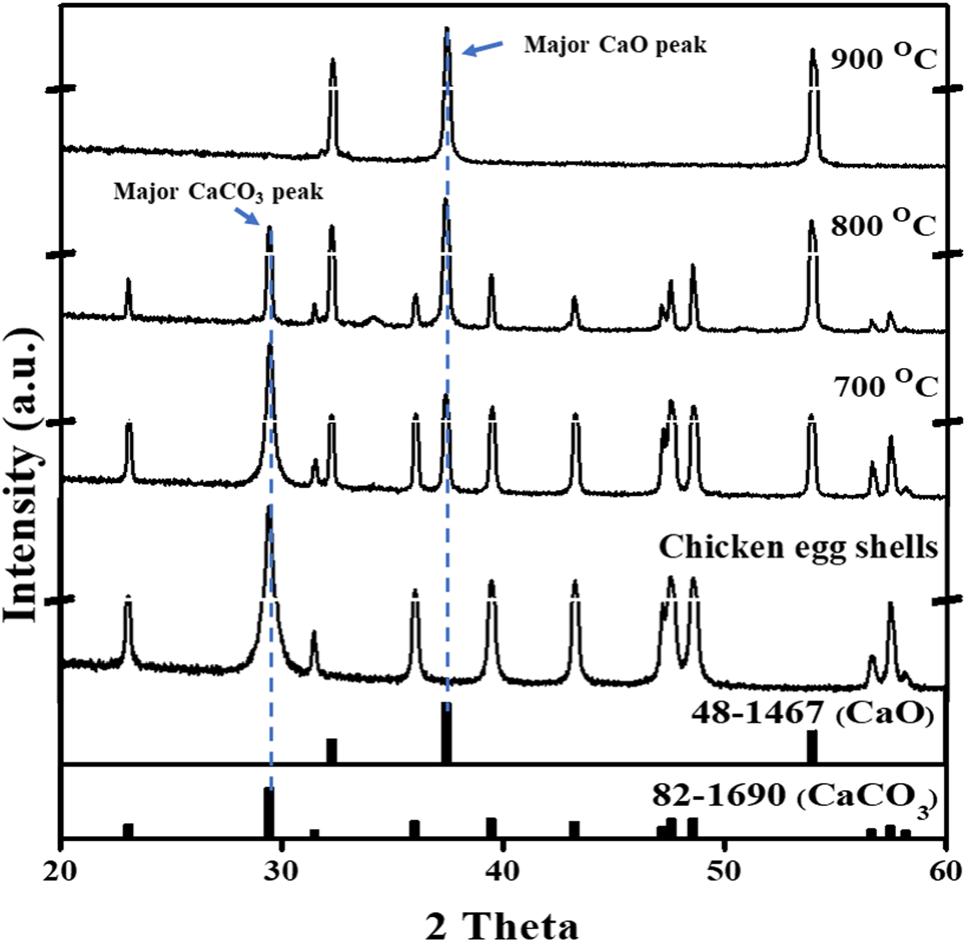
XRD patterns of eggshell waste calcined at different temperatures.

Eggshell waste was calcined at 700 °C, 800 °C, or 900 °C for 2 hours to investigate phase transformations using XRD. The XRD patterns of both raw and calcined eggshell waste samples are presented in Fig. 3. The XRD pattern of the raw eggshell waste had diffraction peaks at 2*θ* = 29.40° (104), 36.0° (110), 39.42° (113), 43.2° (202), and 47.50° (116), corresponding to the crystallographic planes of calcium carbonate (CaCO_3_) based on the Joint Committee on Powder Diffraction Standards (JCPDS no. 82-1690). These results aligned with the findings of Lanzón *et al.*,^18^ who identified calcite (CaCO_3_) as the predominant phase in eggshell waste.

Following calcination at 700 °C, the eggshell waste appeared dark, indicating partial thermal decomposition and the presence of residual organic matter. At 800 °C, the sample appeared as a mixture of dark and white regions, suggesting further decomposition of CaCO_3_ while still retaining a large amount of calcite. The major crystalline phase in the 700 °C and 800 °C samples was identified as calcite. At 900 °C, the eggshell waste appeared completely white, signifying full thermal decomposition. The XRD pattern of the 900 °C calcined sample displayed characteristic peaks at 2*θ* = 32.20°, 37.34°, and 53.85°, corresponding to calcium oxide (CaO) based on JCPDS no. 37-1497. This indicated that CaCO_3_ was almost completely converted to CaO after calcination at 900 °C for 2 hours, in accordance with the thermal decomposition reaction presented in eqn (1).

1 CaCO_3_ (s) → CaO (s) + CO_2_ (g)

These findings agree with Khan *et al.*^20^ and Chen *et al.*,^30^ who reported that the decomposition of CaCO_3_ occurred at temperatures above 850 °C, leading complete transformation into CaO. Furthermore, the total weight loss of the eggshell waste during calcination was 47.85%, which was closely consistent with the 46.43% weight loss reported by Chen *et al.*^30^ This suggested that organic matter in the eggshell waste was fully decomposed, with carbonaceous material converted into CO_2_ gas instead of remaining as char residues. Based on these results, calcination at 900 °C for 2 hours was selected as the optimal condition for further synthesis of CaO_2_, as it ensured the complete decomposition of CaCO_3_ into reactive CaO while minimizing residual organic impurities.

**Fig. 4:**
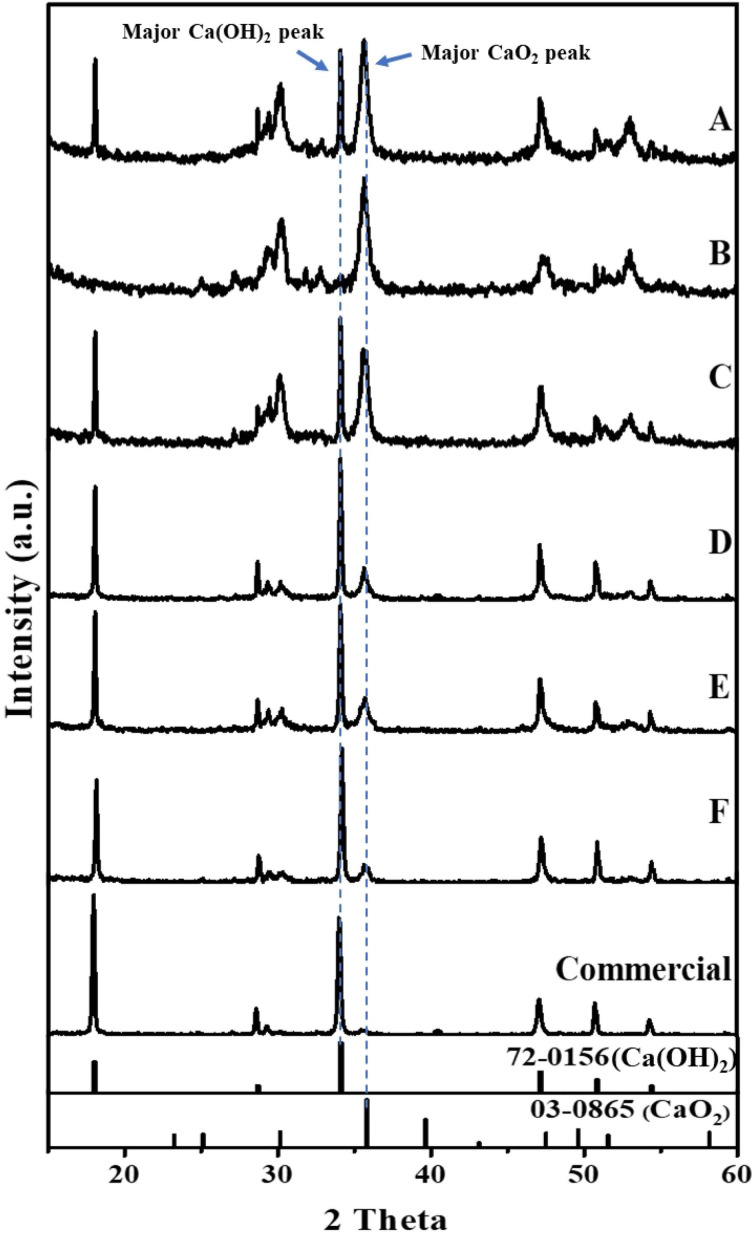
XRD patterns of synthesized CaO_2_ composites at different H_2_O_2_ concentrations. A (H_2_O_2_ 25%, mole ratio 1 : 1), B (H_2_O_2_ 30%, mole ratio 1 : 1), C (H_2_O_2_ 35%, mole ratio 1 : 1), D (H_2_O_2_ 25%, mole ratio 7 : 1), E (H_2_O_2_ 30%, mole ratio 8.5 : 1), F (H_2_O_2_ 35%, mole ratio 10 : 1).

As shown in Fig. 4, the XRD pattern of synthesized CaO_2_ for composite B had characteristic peaks at 2*θ* = 30.27°, 35.59°, and 47.30°, which matches well with the reference pattern for CaO_2_ (JCPDS no. 03-0865). In contrast, the XRD patterns of other samples showed a mixture of both the CaO_2_ and Ca(OH)_2_ phases, indicating incomplete conversion of calcium hydroxide. However, the phase fraction of Ca(OH)_2_ in commercial CaO_2_ was 95.11%, while that of CaO_2_ was 4.89%. The reactions involved in CaO_2_ synthesis are presented in eqn (2) and (3).

2 CaO (s) + H_2_O (l) → Ca(OH)_2_ (s)

3 Ca(OH)_2_ (s) + H_2_O_2_ (aq) → CaO_2_ (s) + 2H_2_O (l)

The percentage phase fraction of CaO_2_ in each sample was calculated and is summarized in Table 2. Moreover, the preparation of CaO_2_ from eggshell waste allows better control of the CaO_2_ phase fraction than commercial CaO_2_, leading to improved catalytic performance. This combination of sustainability, cost-effectiveness, and enhanced material properties highlights the novelty of the present work.

**Table 2:** Phase fraction in synthesized CaO_2_ composites at different H_2_O_2_ concentrations

Composite	Concentration of H_2_O_2_ (%)	Molar ratio Ca(OH)_2_ : H_2_O_2_	Phase fraction (%)	
			CaO_2_	Ca(OH)_2_
A	25	1 : 1	52.00	48.00
B	30	1 : 1	84.23	15.77
C	35	1 : 1	43.26	56.74
D	25	7 : 1	19.14	80.86
E	30	8.5 : 1	21.84	78.16
F	35	10 : 1	12.70	87.30
Commercial	—	—	4.89	95.11

##### 3.1.1.1. Effect of H_2_O_2_ concentration on CaO_2_ formation

**Fig. 5:**
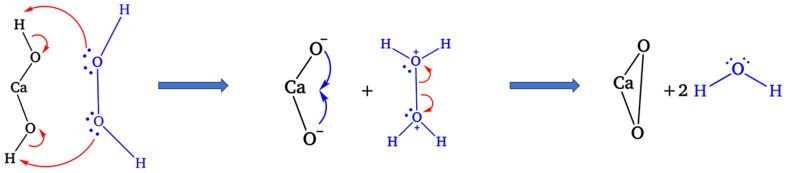
Proposed mechanism of CaO_2_ synthesis derived from H_2_O_2_ and Ca(OH)_2_.

According to Table 2, for composites A, B, and C, where the Ca(OH)_2_ : H_2_O_2_ molar ratio was fixed at 1 : 1, an increase in H_2_O_2_ concentration from 25% to 30% led to a rise in the CaO_2_ phase fraction from 52.00% to 84.23%. However, further increasing the H_2_O_2_ concentration to 35% resulted in a decrease in the CaO_2_ phase fraction to 43.26%. This behavior can be explained by the Lewis base nature of H_2_O_2_, which possesses two lone pairs of electrons on its oxygen atoms. When H_2_O_2_ was introduced at 25% (composite A) and 30% (composite B), lone pair electrons from H_2_O_2_ readily attracted H^+^ from Ca(OH)_2_, facilitating the formation of CaO_2_ according to eqn (3), with water (H_2_O) as a by-product, as illustrated in Fig. 5. However, at a 35% H_2_O_2_ concentration (composite C), the solution pH decreased considerably. The pH of the reaction mixtures decreased with increasing H_2_O_2_ concentration, measured as 12.2 for composite A (25% H_2_O_2_), 11.8 for composite B (30% H_2_O_2_), and 11 for composite C (35% H_2_O_2_). These pH values provide quantitative support for the observed decline in the CaO_2_ phase fraction at higher H_2_O_2_ concentrations, in agreement with the chemical reasoning based on the Lewis base behavior of H_2_O_2_ and proton interactions. According to Hata *et al.*,^31^ increasing the H_2_O_2_ concentration from 0.1% to 1% led to a pH drop from 12.5 to 11.6, approaching the p*K*_a_ of H_2_O_2_. When excessive H_2_O_2_ was added, the increase in H^+^ ions resulted in stronger electrostatic interactions between the H^+^ and the oxygen atoms of Ca(OH)_2_, reducing the attractive force between H_2_O_2_ and Ca(OH)_2_. This hindered the formation of CaO_2_, leading to a lower CaO_2_ phase fraction despite the higher H_2_O_2_ concentration.

##### 3.1.1.2. Effect of molar ratio on CaO_2_ formation

Variations in the Ca(OH)_2_ : H_2_O_2_ molar ratio from 1 : 1 to 7 : 1, 8.5 : 1, and 10 : 1 affected the crystallization of CaO_2_ and the phase fraction of CaO_2_ and Ca(OH)_2_, as shown in Table 2. The observed variations in the Ca(OH)_2_ and CaO_2_ phase fractions could be primarily attributed to the crystallization process and the reaction kinetics governing CaO_2_ formation. The formation of CaO_2_ occurred through the reaction of Ca(OH)_2_ with H_2_O_2_, where an optimal balance between reactant concentrations was necessary to maximize CaO_2_ yield. At a lower Ca(OH)_2_ : H_2_O_2_ molar ratio (1 : 1), a higher fraction of CaO_2_ was formed due to the sufficient availability of H_2_O_2_ to facilitate complete conversion. However, as the molar ratio increased, the amount of unreacted Ca(OH)_2_ also rose, leading to a progressive decline in the CaO_2_ phase fraction. This trend indicates that excess Ca(OH)_2_ did not contribute to additional CaO_2_ formation but instead remained as a residual phase.

Furthermore, the crystallization dynamics suggested that the solubility and reactivity of Ca(OH)_2_ influenced its interaction with H_2_O_2_. Excess Ca(OH)_2_ likely resulted in increased particle aggregation, reducing the effective surface area available for reaction. Additionally, a higher Ca(OH)_2_ concentration may have shifted the reaction equilibrium, hindering the complete transformation into CaO_2_. These findings align with previous studies on CaO_2_ synthesis mechanisms, emphasizing the importance of precise reactant ratio control to optimize phase purity and yield.^31,32^

#### 3.1.2. Fourier-transform infrared spectroscopy analysis

**Fig. 6:**
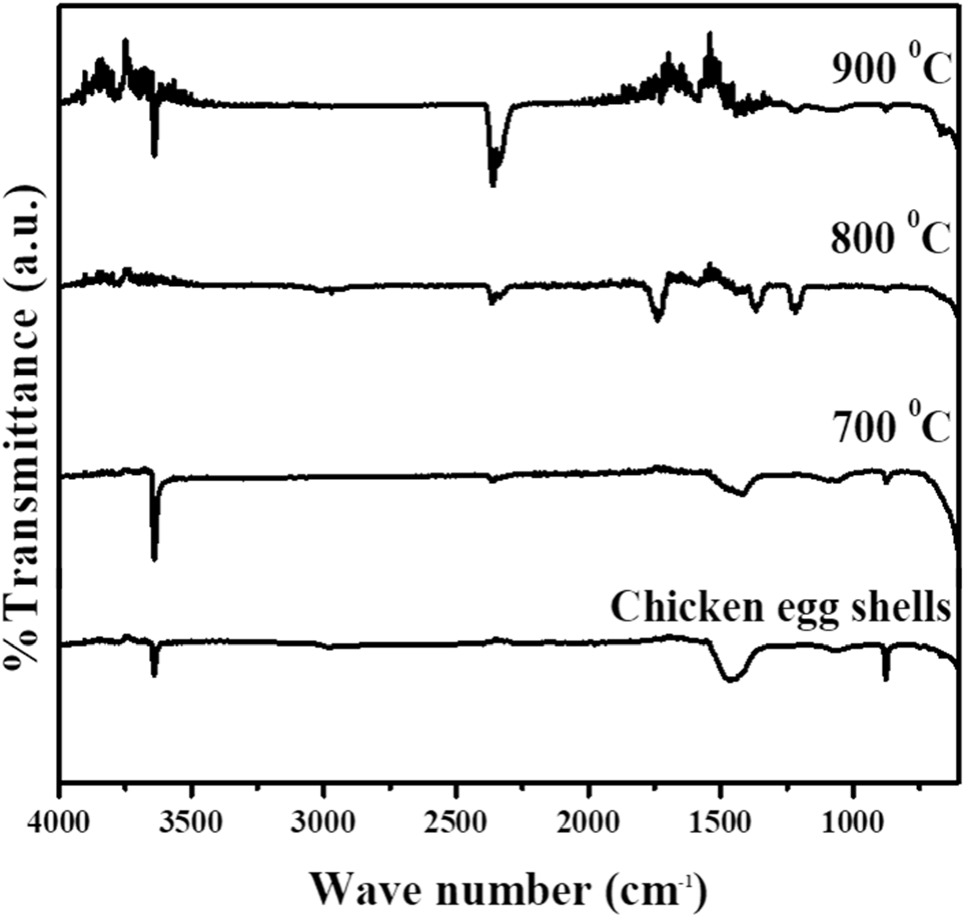
Fourier-transform infrared spectroscopy spectra of eggshell waste and calcined eggshell waste at different temperatures.

FTIR analysis was performed to identify the functional groups present in the eggshell waste, calcined eggshell waste, and synthesized CaO_2_, as shown in Fig. 6. For the eggshell waste, the FTIR spectrum confirmed characteristic carbonate (CO_3_^2−^) functional groups. The absorption band at 870 cm^−1^ corresponded to out-of-plane bending vibrations of CO_3_^2−^, while the band at 1400 cm^−1^ was attributed to asymmetric stretching vibrations of CO_3_^2−^, consistent with the findings reported by Lanzón *et al.*^18^ Upon increasing the calcination temperature to 700 °C and 800 °C, the intensity of the CO_3_^2−^ bands decreased, indicating partial decomposition of CaCO_3_ into CaO. At 900 °C, the carbonate peaks were nearly absent, suggesting that most of the CaCO_3_ had decomposed into CaO, with only minor spectral noise remaining. The absorption band at 3645 cm^−1^ was associated with the presence of hydroxyl (OH) groups, indicating surface hydration of the calcined samples.

**Fig. 7:**
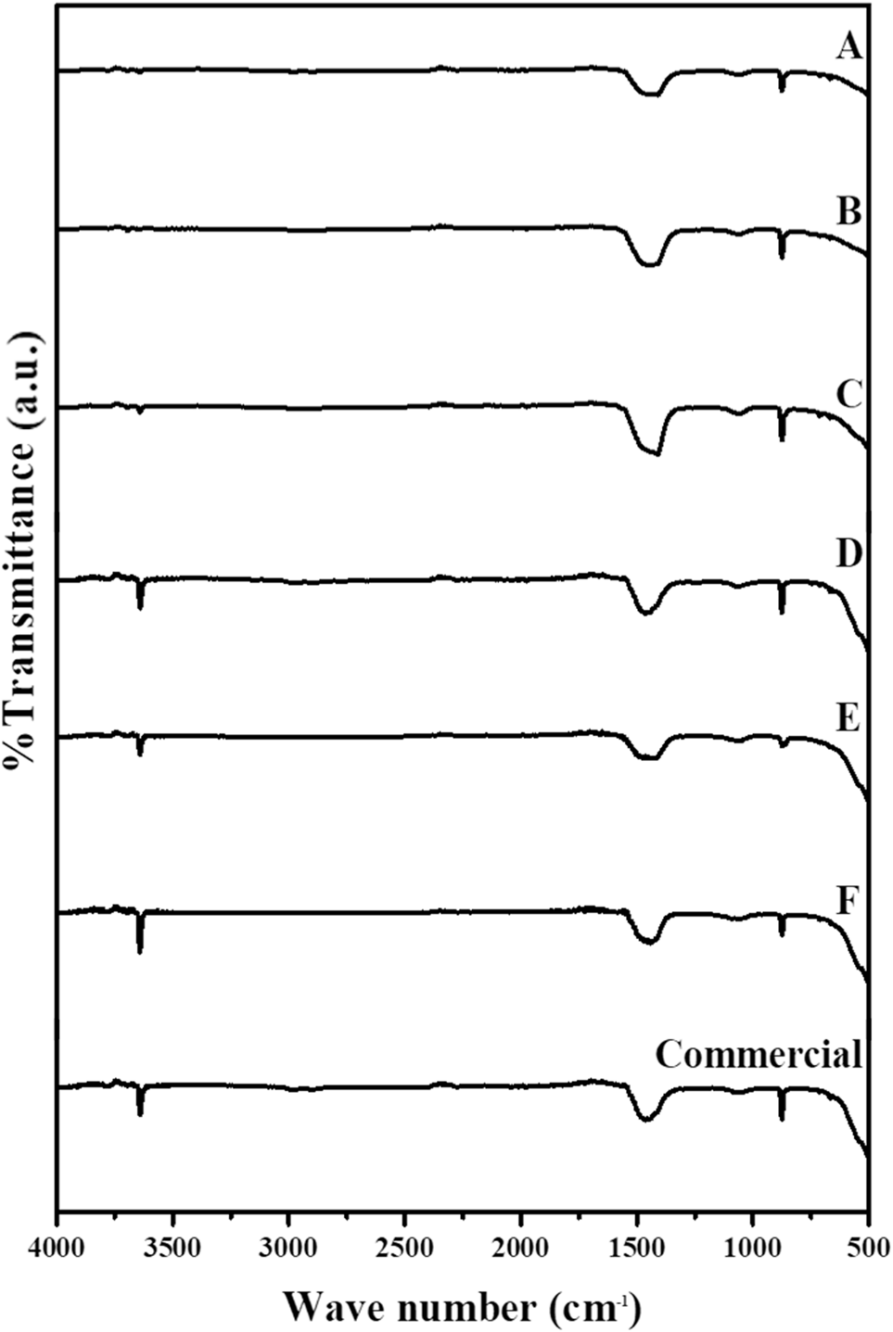
Fourier-transform infrared spectroscopy spectra of synthesized CaO_2_ composites (see Table 1) at different H_2_O_2_ concentrations: A (H_2_O_2_ 25%, mole ratio 1 : 1), B (H_2_O_2_ 30%, mole ratio 1 : 1), C (H_2_O_2_ 35%, mole ratio 1 : 1), D (H_2_O_2_ 25%, mole ratio 7 : 1), E (H_2_O_2_ 30%, mole ratio 8.5 : 1), F (H_2_O_2_ 35%, mole ratio 10 : 1).

For the synthesized CaO_2_, as shown in Fig. 7, the FTIR spectrum showed characteristic O–Ca–O vibrations at 1482 cm^−1^ and 1415 cm^−1^, while the O–O bond of the CaO_2_ molecule was detected at 866 cm^−1^.^34^ These peaks closely matched the spectral features of commercial CaO_2_, including the O–O vibration at 871 cm^−1^ and the O–Ca–O stretching at 1414.8 cm^−1^, confirming the successful synthesis of CaO_2_. The broad peak at 3645 cm^−1^ was attributed to hydroxyl groups, likely from surface hydration.

Moreover, the gradual loss of CO_3_^2−^ bands with increasing calcination temperature indicates the thermal decomposition of CaCO_3_ to CaO. The formation of O–Ca–O vibrations in the synthesized CaO_2_ confirms the peroxide structure, while the O–O vibration at 866 cm^−1^ verifies successful incorporation of the peroxide bond. The broad band at 3645 cm^−1^ in both calcined eggshell and CaO_2_ samples is attributed to surface-adsorbed water. Overall, the FTIR spectra confirm that thermal treatment and synthesis effectively converted eggshell waste into functional CaO_2_ with characteristic chemical bonds.

#### 3.1.3. Scanning electron microscopy analysis

**Fig. 8:**
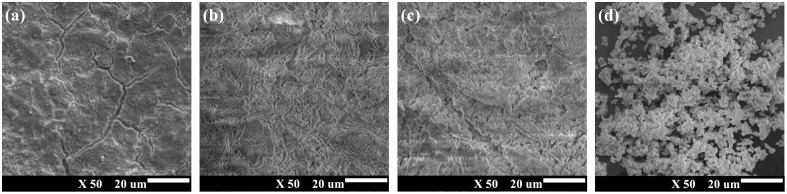
Scanning electron microscopy images of eggshell waste calcined at different temperatures (a) eggshell waste, (b) 700 °C, (c) 800 °C, and (d) 900 °C.

The SEM micrographs provided insights into the morphological transformations of the eggshell waste before and after calcination at different temperatures, as illustrated in Fig. 8. The SEM images of the raw eggshell waste revealed a relatively flat and compact surface morphology. However, after calcination at 900 °C, the surface became much more fragmented and porous, a transformation attributed to the thermal decomposition of calcium carbonate (CaCO_3_) and the subsequent release of carbon dioxide (CO_2_). This structural transformation resulted in a significant reduction in particle size and an increase in pore volume, thereby enhancing the specific surface area and reactivity of the materials.

**Fig. 9:**
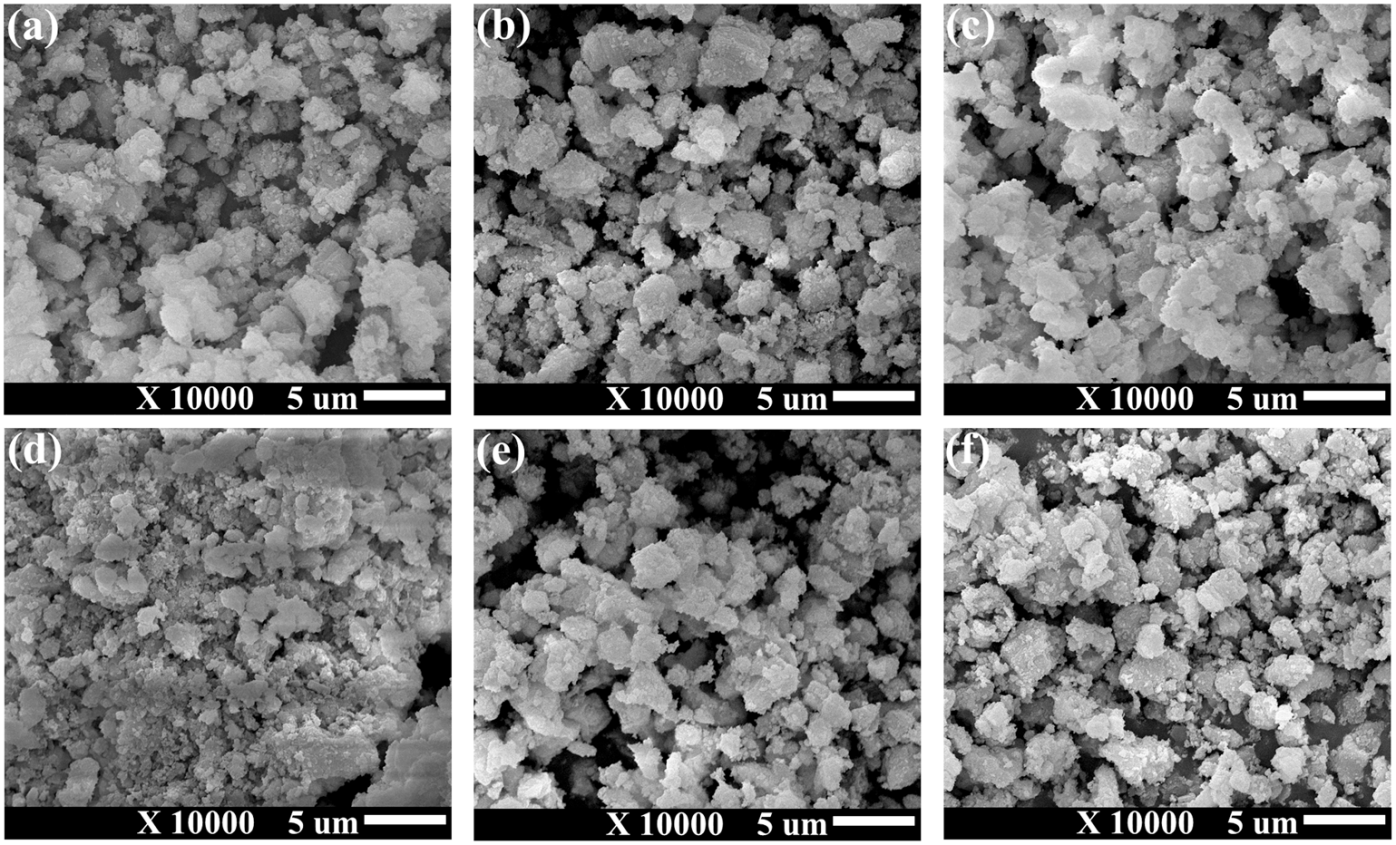
Scanning electron microscopy images of synthesized CaO_2_ under varying synthesis conditions, defined in Table 1 (a) composite A, (b) composite B, (c) composite C, (d) composite D, (e) composite E, and (f) composite F.

Fig. 9 presents the SEM images of the synthesized CaO_2_ composites obtained at different H_2_O_2_ concentrations. The SEM analysis confirmed the successful formation of CaO_2_ particles through the reaction of Ca(OH)_2_ with H_2_O_2_. However, notable agglomeration of CaO_2_ particles was observed, which was likely due to the inherently high surface energy of CaO_2_ particles.^20^ This agglomeration phenomenon may have important implications for catalytic performance, stability, and the controlled-release properties of CaO_2_ in further practical applications.

### 3.2. Study of oxytetracycline degradation efficiency and reaction kinetic

A series of ozonation experiments were conducted at pH 7 to evaluate the efficacy of synthesized CaO_2_ as a catalyst in catalytic ozonation for the degradation of OTC. Each synthesized CaO_2_ composite (A–F) was applied at a dosage of 3 g L^−1^, and the reaction was monitored at various retention times (0, 5, 10, 15, 30, and 60 min). The performance in OTC degradation was monitored and the kinetic data of catalytic ozonation were compared with sole ozonation. Based on the results (Table 3), combining the synthesized CaO_2_ composites using ozonation considerably enhanced OTC removal efficiency, achieving up to 100%, compared to sole ozonation (85.72%). To our knowledge, catalytic ozonation using waste-derived CaO_2_ has not been reported. In this study, CaO_2_ synthesized from eggshell waste achieved complete OTC removal (100%), compared to 91.5% degradation reported by Li *et al.*^35^ using commercial CaO_2_ with O_3_ after 30 min. In particular, composite B, which contained a higher percentage of CaO_2_, showed superior OTC degradation efficiency. In contrast, composite D had a lower removal efficiency (87.86%) than the others due to its lower CaO_2_ fraction. This finding confirmed the major role of CaO_2_ in enhancing OTC degradation during catalytic ozonation by generating OH˙ radicals that had a higher oxidation potential than ozone. These results were consistent with the findings by Giler-Molina *et al.*,^27^ who reported the catalytic effectiveness of CaO_2_ in advanced oxidation processes.

**Table 3:** Pseudo-first-order rate constants (*k*_obs_) for oxytetracycline degradation using different synthesized CaO_2_ samples at pH 7 (catalyst dosage = 3 g L^−1^)^a^

Sample	% removal at 60 min	*k*_obs_ (min^−1^)	*R* ^2^
Sole ozonation	85.72	0.0365	0.9820
A	100.00	0.0421	0.9666
B	100.00	0.1152	0.9142
C	100.00	0.0605	0.9782
D	100.00	0.0371	0.9928
E	100.00	0.0796	0.9536
F	87.86	0.0493	0.9776

a *R* ^2^ = coefficient of determination. See Table 1 for details of components of composites A–F.

Based on the kinetic study, the reaction of OTC degradation by both catalytic ozonation and sole ozonation fit well with a pseudo-first-order kinetic model. According to Table 3, the observed rate constant (*k*_obs_) for sole ozonation was 0.0365 min^−1^. In contrast, the *k*_obs_ values for catalytic ozonation using CaO_2_ composites were consistently higher than for sole ozonation, indicating that the CaO_2_ synthesized from the eggshell waste had considerable catalytic potential in ozonation. This enhancement in degradation efficiency could be attributed to the increased production of hydroxyl radicals (OH˙), which have a higher oxidation potential (2.80 V) than ozone alone (2.07 V). Among the synthesized CaO_2_ composites, composite B had the highest *k*_obs_ value (0.1152 min^−1^), surpassing all other composites. This superior catalytic performance could be attributed to the higher CaO_2_ phase fraction in composite B, which facilitated more efficient OH˙ generation, thereby accelerating OTC degradation.

At pH 7, ozone reacts *via* two primary pathways: (1) direct oxidation, in which ozone itself degrades OTC; and (2) indirect oxidation, where ozone is decomposed to generate hydroxyl radicals (OH˙), which initiate secondary oxidation reactions. The presence of CaO_2_ enhances OH˙ generation *via* a series of mechanisms. CaO_2_ slowly releases H_2_O_2_ and O_2_, as shown in eqn (4) and (5).

4 CaO_2_ + 2H_2_O → H_2_O_2_ + Ca(OH)_2_

5 2H_2_O_2_ → 2H_2_O + O_2_

Then, the hydroxyl radical (OH˙) is generated through the reaction between H_2_O_2_ and O_3_, as shown in eqn (6)–(10).

6 H_2_O_2_ + H_2_O → HO_2_^−^ + H_3_O^+^

7 O_3_ + HO_2_^−^ → HO_2_^−^ + O_2_

8 O_3_ + HO_2_^−^ → OH˙ + O_2_^−^ + O_2_

9 O_3_ + O_2_^−^ → O_3_^−^ + O_2_

10 O_3_^−^ + H_2_O → OH˙ + O_2_ + OH^−^

Furthermore, the dissociation of Ca(OH)_2_ releases OH^−^ ions, leading to an increase in pH, which in turn promotes the formation of hydroperoxyl radicals (HO_2_˙), as illustrated in eqn (11) and (12).^6,8,33^

11 Ca(OH)_2_ → Ca^2+^ + 2OH^−^

12
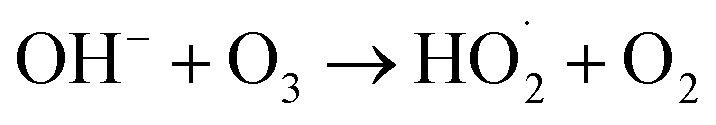


These findings suggest that the CaO_2_ synthesized from eggshell waste not only serves as an efficient catalyst for OTC degradation *via* ozonation but also provides a promising and sustainable alternative to commercial CaO_2_ catalysts.

## 4. Conclusions

CaO_2_ was successfully synthesized from eggshell waste and its effectiveness was demonstrated as a catalyst in the ozonation process for the degradation of oxytetracycline (OTC). The optimal calcination temperature was 900 °C for converting CaCO_3_ from the eggshell waste into CaO, ensuring complete phase transformation. For CaO_2_ synthesis, the optimum Ca(OH)_2_-to-H_2_O_2_ molar ratio was 1 : 1, with an H_2_O_2_ concentration of 30%, yielding the highest CaO_2_ phase fraction (84.23%). The synthesized CaO_2_ composites combined with ozonation considerably enhanced OTC degradation efficiency, achieving up to 100% removal, compared to sole ozonation (85.72%).

Based on the kinetic analysis, the value of the pseudo-first-order rate constant (*k*_obs_) was 0.1152 min^−1^, for catalytic ozonation using composite B as a catalyst, which was considerably higher than that of sole ozonation (0.0365 min^−1^) and even greater than that of commercial CaO_2_ (0.0880 min^−1^). This enhanced oxidation performance was primarily attributed to the higher CaO_2_ phase fraction in composite B, which facilitated efficient generation of hydroxyl radicals (OH˙), providing a higher oxidative potential.

In summary, CaO_2_ synthesized from eggshell waste with a high fraction of CaO_2_ demonstrates significant potential as an effective catalyst for catalytic ozonation processes. This study highlighted the feasibility of using waste-derived CaO_2_ as a sustainable and efficient catalyst for advanced oxidation processes.

## Author contributions

Apiradee Sukmilin: conceptualization; data curation; formal analysis; funding acquisition; project administration; writing – original draft and manuscript editing. Piyapong Pankaew: data curation; XRD, SEM and FTIR analysis; characterization; writing. Jaroenporn Chokboribal: FTIR analysis and graphical drawing. Chalor Jarusutthirak: conceptualization and manuscript editing.

## Conflicts of interest

The authors declare that they have no known competing financial interests or personal relationships that could have appeared to influence the work reported in this paper.

## Data Availability

Data underlying this study are available from the corresponding author on reasonable request.
